# Detachment of surface membrane invagination systems by cationic amphiphilic drugs

**DOI:** 10.1038/srep18536

**Published:** 2016-01-04

**Authors:** Sangar Osman, Kirk A. Taylor, Natalie Allcock, Richard D. Rainbow, Martyn P. Mahaut-Smith

**Affiliations:** 1 Department of Molecular and Cell Biology, University of Leicester, Leicester, UK, LE1 9HN.; 2 Department of Cardiovascular Sciences, University of Leicester, Leicester, UK, LE1 9HN; 3 Centre for Core Biotechnology Services, University of Leicester, Leicester, UK, LE1 9HN

## Abstract

Several cell types develop extensive plasma membrane invaginations to serve a specific physiological function. For example, the megakaryocyte demarcation membrane system (DMS) provides a membrane reserve for platelet production and muscle transverse (T) tubules facilitate excitation:contraction coupling. Using impermeant fluorescent indicators, capacitance measurements and electron microscopy, we show that multiple cationic amphiphilic drugs (CADs) cause complete separation of the DMS from the surface membrane in rat megakaryocytes. This includes the calmodulin inhibitor W-7, the phospholipase-C inhibitor U73122, and anti-psychotic phenothiazines. CADs also caused loss of T tubules in rat cardiac ventricular myocytes and the open canalicular system of human platelets. Anionic amphiphiles, U73343 (a less electrophilic U73122 analogue) and a range of kinase inhibitors were without effect on the DMS. CADs are known to accumulate in the inner leaflet of the cell membrane where they bind to anionic lipids, especially PI(4,5)P_2_. We therefore propose that surface detachment of membrane invaginations results from an ability of CADs to interfere with PI(4,5)P_2_ interactions with cytoskeletal or BAR domain proteins. This establishes a detubulating action of a large class of pharmaceutical compounds.

Adaptation of cellular membrane morphology allows specialized cells to perform dedicated functions. For example, photoreceptors develop surface membrane invaginations or internal membrane disks to increase the surface area for expression of opsins and other proteins, thereby increasing sensitivity to light^1^,^2^,^3^. Another example is the regular invagination of the plasma membrane in striated muscle, known as the transverse (T) tubular system, which plays a key part in excitation:contraction coupling^4^,^5^,^6^,^7^. The arrangement of T tubules, also referred to as the transverse axial tubular system in cardiac muscle, promotes efficient release of Ca^2+^ from the sarcoplasmic reticulum leading to activation of the Ca^2+^-dependent contractile machinery^8^. Depending upon the muscle type, this coupling may be via a direct voltage-dependent release mechanism, as in mammalian skeletal muscle^6^, or via a Ca^2+^-dependent release mechanism relying upon initial Ca^2+^ influx through voltage-gated Ca^2+^ channels, as occurs in the heart^9^. In both cases, the close proximity of a dihydropyridine-sensitive voltage-dependent protein on the surface membrane to the ryanodine receptor on the cellular Ca^2+^ stores is crucial. It is not surprising therefore that altered T tubule structure is emerging as a likely contributory factor to heart failure in several disease models^10^,^11^,^12^.

Another important surface invagination system is the demarcation membrane system (DMS) of megakaryocytes, large polyloid marrow cells responsible for producing blood platelets^13^,^14^,^15^,^16^,^17^. An adult human has almost 10^12^ circulating platelets, which have a limited lifespan of 5–9 days^18^. It can therefore be estimated that more than a million platelets must be released every second throughout the body to maintain the normal platelet count. The DMS represents an essential membrane reservoir in support of thrombopoiesis^17^ and consequently diseases that alter the structure of this membrane system lead to thrombocytopenia^19^,^20^. The platelet possesses a similar membrane invagination system known as the open canalicular system (OCS). This also serves as a membrane reservoir, supporting the spreading of platelets over the collagen-rich exposed subendothelial vasculature during the initial stages of haemostasis and thrombosis^21^,^22^,^23^.

Despite their importance, the control and stability of plasma membrane invagination systems is poorly understood. Previous measurements of the megakaryocyte DMS have mainly relied on EM studies which are time-consuming, difficult to quantify and require measurements in fixed tissues^19^,^24^. Here, we have developed a straightforward assay of the megakaryocyte DMS using confocal microscopy and an extracellular impermeant indicator. We report the ability of a range of cationic amphiphilic drugs (CADs), including clinically used phenothiazines, to collapse the DMS and also induce loss of tubules in ventricular cardiac myocytes and platelets. The results provide insight into the mechanisms that regulate stability of membrane invagination systems.

## Results

### Disruption of the megakaryocyte DMS by the widely used calmodulin inhibitor W-7 via a mechanism independent of metalloproteinases

**Figure 1:**
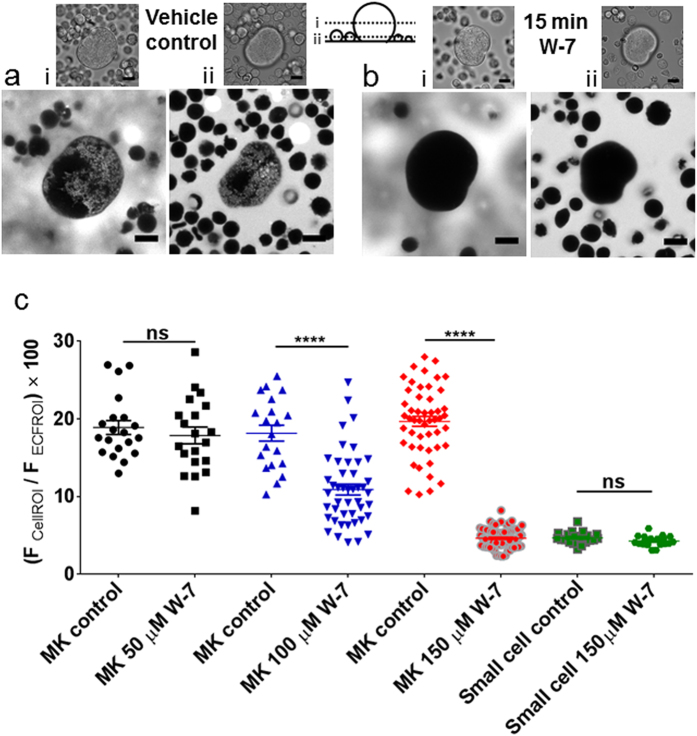
Entry of an extracellular impermeant fluorescent indicator into the megakaryocyte DMS is blocked by W-7. Primary rat marrow cells were incubated with either (**a**) vehicle control (DMSO) or (**b**) 150 μM W-7 for 15 min then exposed to HPTS and confocal fluorescence images collected at two focal planes: (i) mid-way through a megakaryocyte and (ii) mid-way through the centre of most small marrow cells. (**c**) Average HPTS fluorescence within the cell expressed as a percentage of the average extracellular fluorescence (see methods for further details) following 15 min incubation in vehicle or W-7 for megakaryocytes (50, 100 and 150 μM) and small marrow cells (150 μM). Scale bars: 10 μm. Each symbol in this and subsequent figures is the measurement from an individual cell.

To screen a range of compounds for their ability to modify the megakaryocyte DMS, we developed a confocal microscopic assay that assesses the amount of impermeant extracellular indicator (HPTS, 8-​Hydroxypyrene-​1,3,6-​trisulfonic acid, trisodium salt) within the surface-connected tubules of this membrane invagination system (see Fig. 1a). When expressed as a percentage of the average extracellular signal (see methods), the fluorescence within a region of interest drawn around the whole megakaryocyte was in the range 12.98–26.95% (average 18.88 ± 0.89%, n = 20, compared to 4.67 ± 0.03%, n = 20, for small marrow cells (Fig. 1c). The percentage extracellular dye entering the megakaryocyte DMS was heterogeneous (Fig. 1c) but correlated with cell size (Supplementary Fig. S1), as previously observed for capacitance measurements^25^, and therefore reflects the development and quantity of the DMS. Small marrow cells reject the extracellular dye (Fig. 1a, lower confocal plane), due to their lack of membrane invaginations^15^,^25^, therefore, the percentage extracellular indicator measurement reported for these small cells is due to out of focus fluorescence. This assay allowed us to screen a wide range of kinase inhibitors, cytoskeletal modifying reagents and modulators of intracellular Ca^2+^ signalling, most of which had no effect on the percentage dye entering the megakaryocyte DMS (Table S1). However, the compound W-7 (150 μM, 15 min), commonly used as a calmodulin antagonist, completely prevented dye entry into the DMS (Fig. 1b,c). This effect was observed in all megakaryocytes tested (n > 80) and was concentration-dependent (Fig. 1c). W-7 was included in our screen due to its ability to cause ectodomain cleavage of several membrane glycoproteins including GPIb^26^,^27^, a platelet-specific protein which when absent leads to disordered megakaryocyte DMS^19^. However, the timecourse of W-7 action on dye entry into the DMS was maximal after 15 minutes (not shown), which is considerably shorter than that required to cleave GPIb (30–60 min)^27^. In addition, the loss of extracellular dye entry following W-7 treatment was partially reversible after 2 hours wash and not inhibited by the general metalloproteinase inhibitor GM6001 (Supplementary Fig. S2). Moreover, two other reagents that cause ectodomain shedding of membrane glycoproteins, CCCP and N-ethylmaleimide, did not affect the level of HPTS entering the DMS (supplementary Table S2). Taken together, these observations demonstrate that ectodomain cleavage is not involved in the ability of W-7 to perturb the DMS.

### W-7 causes physical separation of the peripheral plasma membrane from the DMS

**Figure 2:**
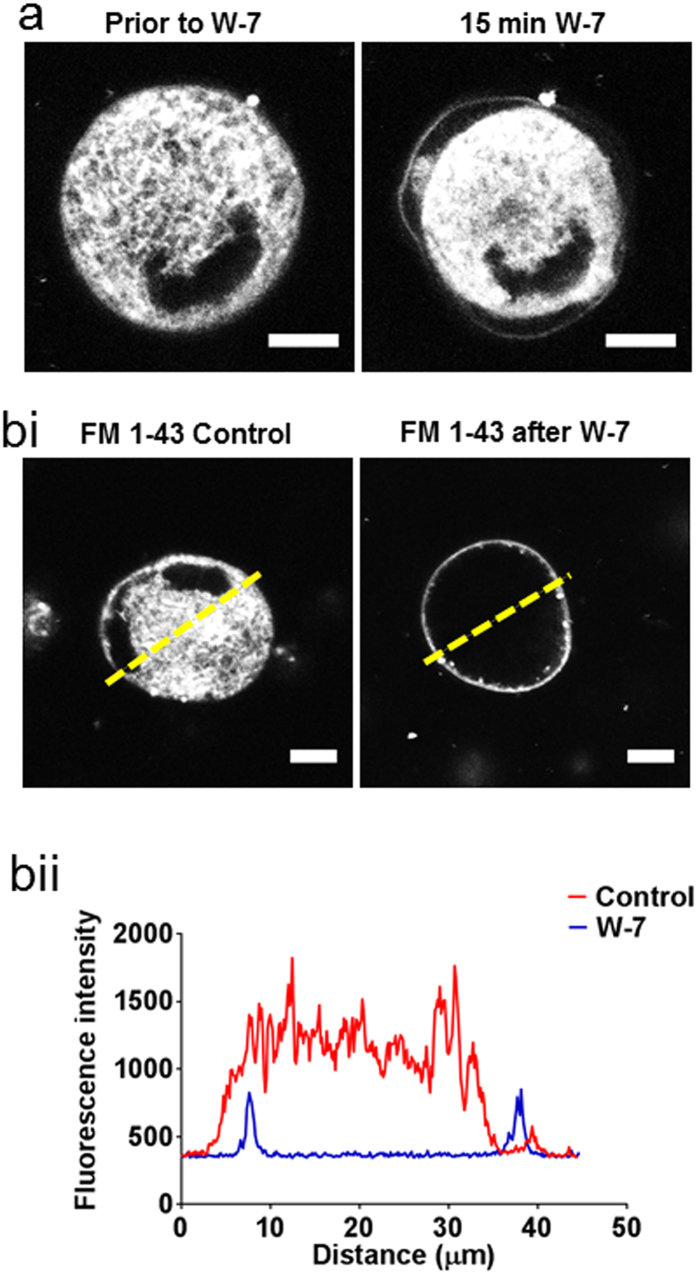
W-7 causes movement of the peripheral plasma membrane away from the underlying DMS. Confocal fluorescence images from megakaryocytes stained with the impermeant membrane indicator FM 1–43. (**a**) Staining pattern for a megakaryocyte before (left panel) and 15 min after (right panel) exposure to 150 μM W-7 (see also supplementary movie S1). (**b**i) Staining pattern for FM 1–43 added 15 min after either DMSO (vehicle control, left image) or 150 μM W-7 (right image). (**b**ii) Fluorescence profile across the lines shown in (**b**i). Scale bars: 10 μm.

**Figure 3:**
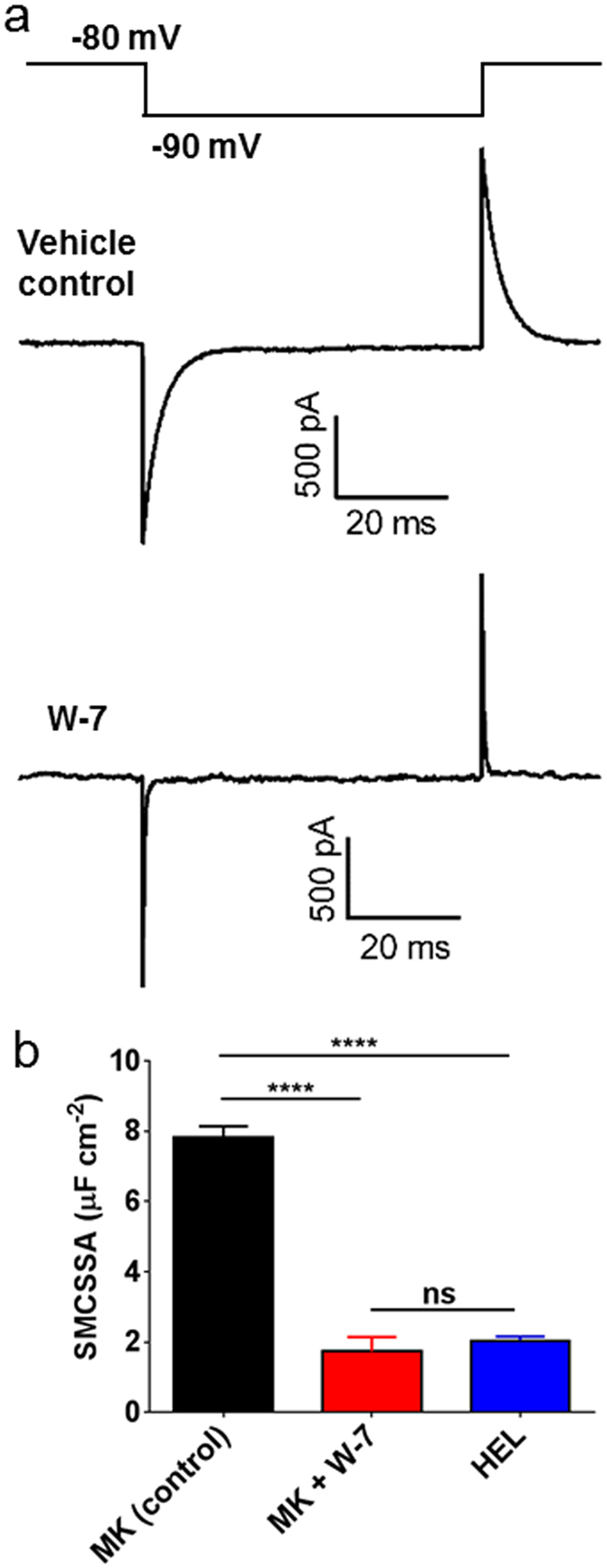
Reduction in megakaryocyte whole cell membrane capacitance by W-7. Whole cell patch clamp measurements of (**a**) Whole cell currents recorded in a megakaryocyte treated for 15 minutes with DMSO (centre trace) or 150 μM W-7 (lower trace) in response to a voltage step from −80 mV to −90 mV (upper trace). (**b**) Average whole cell capacitance derived from the integral of the capacitative currents, normalised to the peripheral surface area, calculated assuming a spherical geometry (SMCSSA: specific membrane capacitance per spherical surface area). Data are from a minimum of four independent experiments for each condition.

**Figure 4:**
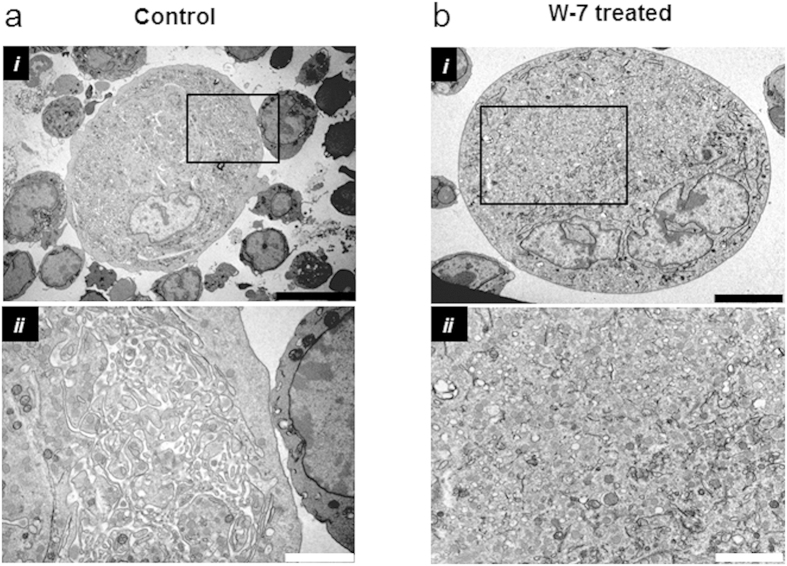
Ultrastructural studies show that W-7 collapses the demarcation membrane system. Representative images acquired by transmission electron microscopy from ultra-thin sections of a megakaryocyte treated for 15 min with (**a**) DMSO (vehicle control) or (**b**) W-7 (150 μM). The higher magnification image in (ii) is from the rectangular area shown in (i) and illustrates typical demarcation membrane system appearance within the extranuclear volume of the control megakaryocyte and its collapse following W-7 treatment. Scale bars: 10 μm in **a**i, 5 μm in **b**i and 2 μm for **a**ii and **b**ii.

In untreated megakaryocytes, the impermeant, reversible membrane indicator FM 1–43 stained the DMS extensively through the extranuclear volume, as shown previously for the irreversible styryl lipophilic dye di-8-ANEPPS^25^. Following treatment with W-7, the peripheral plasma membrane was observed to move away from the underlying stained DMS (Fig. 2a and Supplementary Movie S1). If added after W-7, FM 1-43 only stained the very periphery of the megakaryocyte (Fig. 2b), as expected if the DMS was no longer accessible from the extracellular space. The DMS can also be quantified by membrane capacitance measurements, assessed from the transient capacitative current required to change the membrane voltage over a range that does not activate voltage-gated currents^25^,^28^. W-7 caused a marked reduction in membrane capacitance normalised to the theoretical surface area assuming a spherical geometry; this decreased from 7.8 ± 0.32 in vehicle-treated cells to 1.73 ± 0.41 μF/cm^2^ after 150 μM W-7 (*P *<* 0.0001*, n ≥ 4; Fig. 3). The capacitance after W-7 was indistinguishable from that measured for similar sized HEL cells (2.04 ± 0.12 μF/cm^2^; *P* *>* *0.05*, n = 10, Fig. 3b), a myeloid cell line that displays little or no membrane invaginations^25^. The slightly higher specific membrane capacitance of HEL cells and W-7-treated megakaryocytes compared to that commonly reported for biological membranes (1 μF/cm^2^), likely reflects a small amount of surface membrane blebbing or micovilli^29^. The marked decrease in capacitance, together with the loss of styryl dye staining and extracellular dye access suggests that W-7 causes the DMS to become physically disconnected from the peripheral plasma membrane. This conclusion was further supported by transmission electron microscopy measurements (Fig. 4; representative of 48 and 39 cells in control and W-7-treated samples, respectively). The DMS of a vehicle-treated megakaryocyte had a typical appearance, as widely reported elsewhere^14^,^15^. Extensive interconnected vacuoles were observed throughout the cytoplasm with the exception of an organelle-free peripheral zone under the plasma membrane in mature megakaryocytes (e.g. Fig. 4a). W-7 caused a total collapse of the DMS, leaving only short lengths of double membrane with little space between them (Fig. 4b).

### Detachment of the DMS from the peripheral membrane is a common action of cationic amphiphilic but not anionic amphiphilic drugs

**Figure 5:**
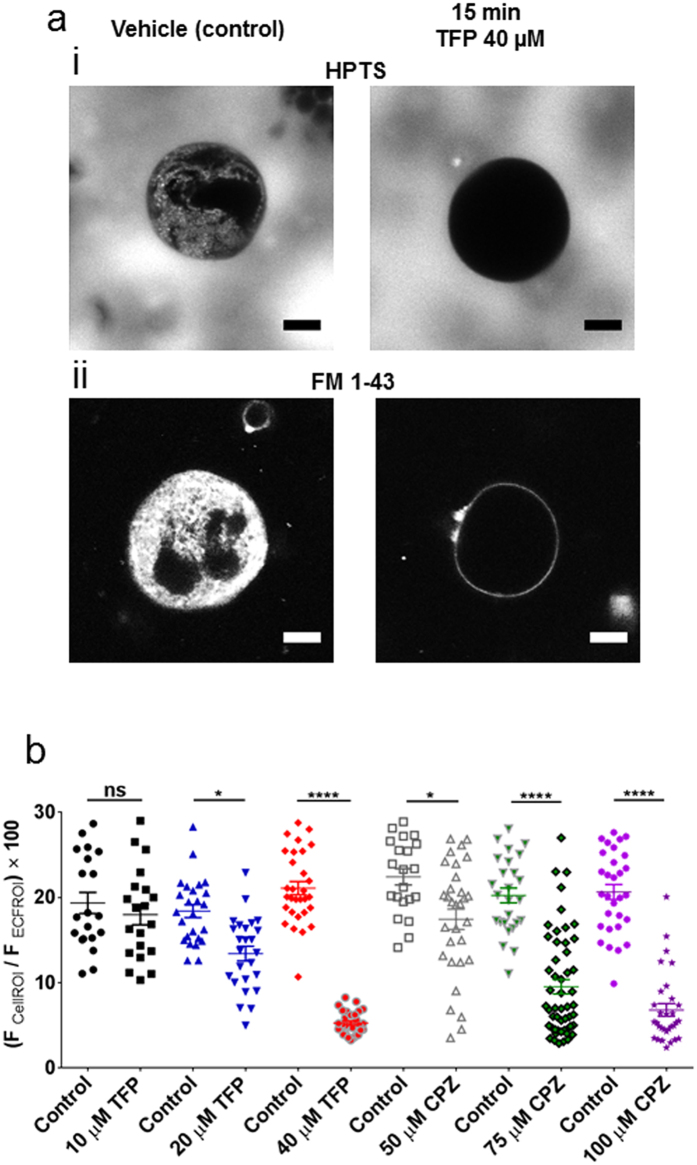
Phenothiazines cause separation of the DMS from the peripheral plasma membrane. (**a**) Confocal fluorescence images from a megakaryocyte under control conditions (left images) and 15 minutes after treatment with trifluoperazine (TFP, 40 μM; right images). The cells were immersed in HPTS (i) or FM 1–43 (ii). (**b**) Percentage HPTS fluorescence within a whole megakaryocyte region of interest compared to the extracellular fluid for different concentrations of TFP and chlorpromazine (CPZ). Measurements from control (DMSO-treated) megakaryocytes were taken for each batch of cells exposed to the different phenothiazine concentration. Scale bars: 10 μm.

W-7 is often employed as a calmodulin antagonist. Another widely used calmodulin antagonist, trifluoperazine, also prevented staining of the megakaryocyte DMS by extracellular impermeant indicators (Fig. 5). Interestingly, trifluoperazine is used clinically as an anti-psychotic reagent, along with chlorpromazine and other phenothiazines, which have been reported to exert marked thrombocytopenia^30^,^31^,^32^. Chlorpromazine caused a similar complete collapse of the DMS (Fig. 5b), suggesting that this is a common action of these anti-psychotic drugs. However, we were unable to interfere with the action of W-7 or TFP using a range of kinase inhibitors, including those that are calmodulin-dependent, or agents that modulate intracellular Ca^2+^ (ionomycin or BAPTA) (supplementary Table S2). Together, these results suggest that a mechanism independent of calmodulin signalling causes the DMS to collapse following treatment with W-7 and a number of phenothiazines.

**Figure 6:**
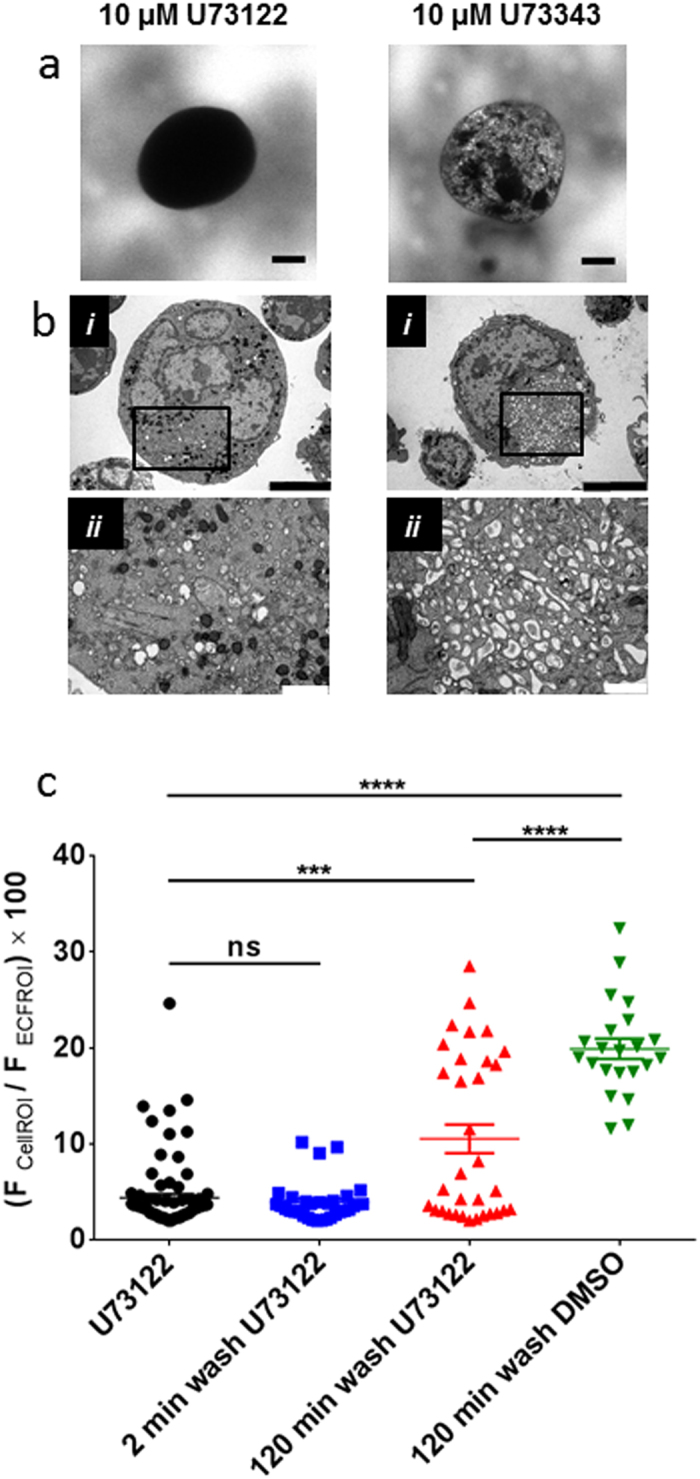
U73122, but not its less electrophilic analogue U73343, cause collapse of the DMS. Confocal fluorescence images in the presence of extracellular HPTS (**a**) and transmission electron microscopy images (**b**) from megakaryocytes exposed for 15 min to 10 μM U73122 (left) or 10 μM U73343 (right). (**b**ii) Shows a higher magnification, higher resolution image of the megakaryocyte in (i), illustrating collapse of the DMS caused by U73122 but not U73343. (**c)** Percentage HPTS fluorescence within megakaryocytes treated with U73122 (10 μM, 15 min) and after a 2 or 120 minute wash period. Scale bars: 10 μm in **a**, 5 μm in **b**i and 1 μm in **b**ii.

Another inhibitor within our screen that prevented extracellular HPTS access to the DMS tubules is the phospholipase-C (PLC) inhibitor, U73122 (1-[6-[[(17β)-3-Methoxyestra-1,3,5(10)-trien-17-yl]amino]hexyl]-1*H*-pyrrole-2,5-dione) (Fig. 6a left panel and 6c). Its ability to collapse the DMS was further demonstrated by transmission electron microscopy studies (Fig. 6bi,ii left panels). Like W-7, the collapse of the DMS by U73122 was slowly reversible (Fig. 6c). A property shared by U73122, W-7, trifluoperazine and other phenothiazines, is that they are all cationic amphiphilic drugs (CADs). These compounds accumulate preferentially in the intracellular leaflet of the plasma membrane due to their attraction to anionic phospholipids^33^. Several other CADs exerted collapse of the DMS, including imipramine, verapamil, propranolol, bupavaciane (Table S3). In contrast, anionic amphipilic drugs such as aspirin, sodium dodecyl sulfate and thiopental, which will accumulate in the outer leaflet of the bilayer^33^,^34^, caused no alteration of HPTS staining (Supplementary Fig. S3; Supplementary Table S3). Further evidence that attraction to charged lipids underlies the ability of these CADs to induce DMS collapse is the lack of effect on this membrane system of U73343 (1-[6-[[(17*β*)-3-Methoxyestra-1,3,5(10)-trien-17-yl]amino]hexyl]-2,5-pyrrolidinedione) (Fig. 6a,b, right panels). U73343 is an analogue of U73122 with a substitution that reduces its electrophilic nature and ability to interfere with phosphatidylinositol (PI) and PI(4,5)bisphosphate (PI(4,5)P_2_) turnover^35^.

### Cationic amphiphilic compounds also cause detubulation in cardiac myocytes and platelets

**Figure 7:**
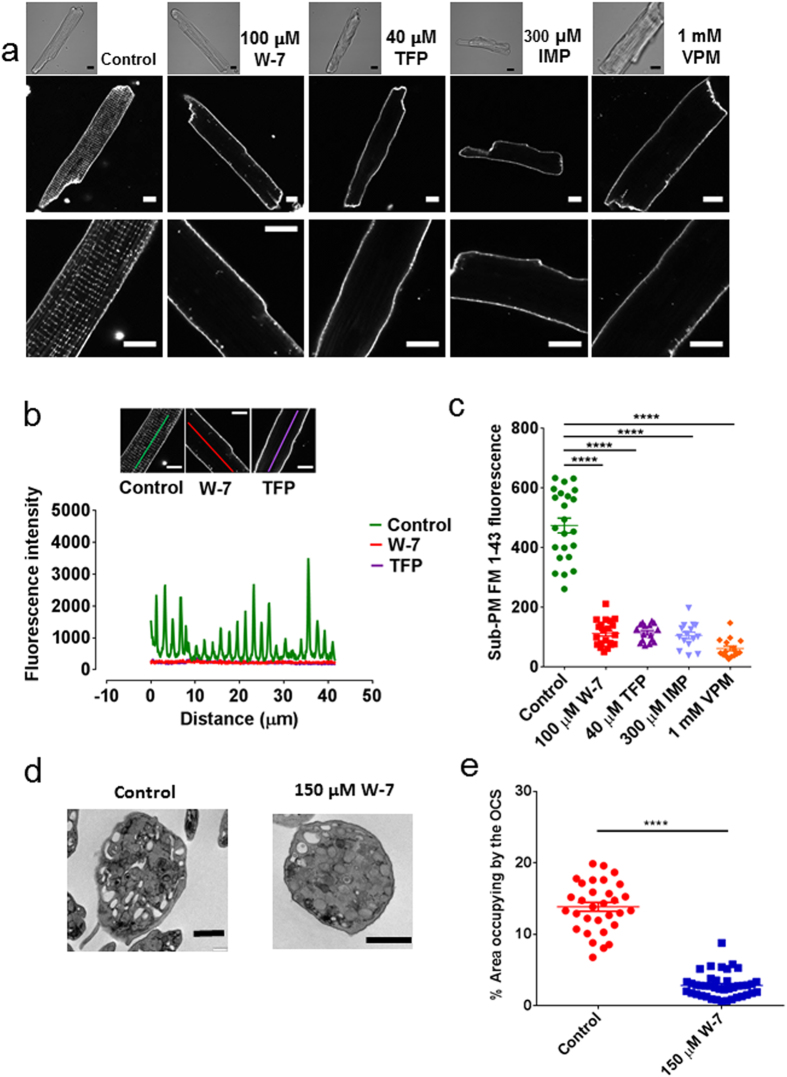
Cationic amphiphilic drugs cause loss of cardiac myocyte T tubules and the platelet open canalicular system. (**a**) FM 1–43 staining of cardiac myocytes under control (DMSO-treated) conditions and after 15 min treatment with W-7, TFP, imipramine (IMP) or verapamil (VPM) at the concentration stated. (**b**) Line fluorescence profile for a control, W-7 and TFP-treated myocyte (see inset images for line placement). (**c**) Average FM 1–43 fluorescence within a region of interest drawn around the periphery of the cell but excluding the peripheral plasma membrane (Sub-PM FM 1–43 fluorescence). (**d**) Sample TEM sections through human platelets treated with vehicle or W-7 (150 μM) for 15 min. (**e**) Percentage cytoplasmic area occupied by vacuolar space; each symbol is from a different platelet. Scale bars are 10 μm in (**a**,**b**) and 1 μm in (**d**).

We were also interested in whether reagents that collapse the DMS exert similar effects on specialised membrane invagination systems in other cell types. Although cardiac myocyte T tubules have a similar or slightly larger diameter than the DMS^8^,^15^,^36^, they occupy far less volume within the whole cell compared to in the megakaryocyte, yielding a weaker signal when filled with an extracellular indicator such as HPTS. However, FM 1-43 generated a clear staining pattern of this regular membrane invagination system (Fig. 7a), as reported previously^37^. A range of CADs that collapsed the DMS (W-7, trifluoperazine, imipramine and verapamil) also caused a marked reduction of FM 1–43 sub-plasma membrane staining of myocytes (Fig. 7a–c; see Supplementary Movies S2,S3,S4,S5,S6 for the z-series), indicating that they detach this muscle membrane invagination system from the cell surface. In contrast to the megakaryocyte DMS, this action was not readily reversible following a 2 hour wash (Supplementary Fig. S4). Due to the small size of platelets and the pronounced shape change that they display on glass surfaces, we resorted to electron microscopy to analyse effects of CADs on the OCS. The OCS represents the main vacuolar space within platelets^21^,^22^, which virtually disappeared after a 15 minute incubation in 150 μM W-7 (see representative images in Fig. 7d). When expressed as a percentage of the entire area of each platelet, the vacuolar content was reduced from 13.84 ± 0.62% (n = 31) in control samples to 2.84 ± 0.26% (n = 41) after W-7 (P < 0.0001) (Fig. 7e).

## Discussion

Cationic amphiphilic drugs (CADs) represent one of the largest classes of pharmaceutical compounds, which are used as anti-depressants, anti-malarial, anti-bacterial, anti-arrhythmic and cholesterol-lowering reagents^38^. They contain a hydrophobic ring structure that promotes membrane permeability and a hydrophilic side chain with a charged cationic amine. Their ability to accumulate in the inner leaflet of the plasma membrane lipid bilayer results from an attraction to asymmetrically distributed anionic phospholipids^33^. With an estimated 3 to 5 negative charges per molecule, PI(4,5)P_2_ is a major target for CAD binding in the membrane^39^,^40^. CADs will also bind strongly to PI(3,4)P_2_, and PI(3,4,5)P_3_, however these are less abundant compared to PI(4,5)P_2_ under resting conditions^40^. PI(4,5)P_2_ plays widespread roles in recruiting proteins to the membrane via electrostatic docking interactions and pleckstrin homology (PH) domains, including scaffolding molecules that link the plasma membrane to the cytoskeleton^40^,^41^. The BAR (Bin-Amphiphysin-Rvs) domain superfamily of proteins bind to PI(4,5)P_2_ through polycationic regions and have diverse roles in cellular architecture and function through their ability to curve membranes^40^,^42^,^43^. Thus, of relevance to our study is the established role for PI(4,5)P_2_ in DMS formation^17^ and the fact that BAR domain proteins are important for biogenesis of both T tubules and the demarcation membrane system. BIN1 (Bridging Integrator 1, also known as amphiphysin 2) is involved in formation or maintenance of T tubules in skeletal^44^ and cardiac^45^ muscle. In addition, individual knock-out of the F-BAR domain proteins, PACSIN2^46^ and CIP4^47^, leads to an altered DMS and thrombocytopenia. PACSIN2 interacts with the underlying cytoskeletal and scaffold protein filamin A^46^, while CIP4 links with the cytoskeleton via Wiskott-Aldrich Syndrome Protein (WASP)^47^. Another possible target for PI(4,5)P_2_ is the submembranous cytoskeletal protein spectrin, which can bind through its PH domain and is also important for DMS formation^48^. We therefore hypothesize that the detubulating action of CADs observed in the present study results from binding to PI(4,5)P_2_ which interferes with the ability of this phosphoinositide to interact directly or indirectly with the cytoskeleton in manner that is regulated by BAR domain proteins. This can explain the W-7-induced movement of the peripheral plasma membrane away from the underlying DMS, along with DMS collapse (Fig. 2a, Supplementary Movie S1). Collapse of the DMS was also observed following treatment with U73122, which is cationic amphiphilic in nature and commonly used as a PLC inhibitor^35^. The precise mechanism whereby U73122 inhibits PLC is unclear, but could also result from its attraction to PI(4,5)P_2_ in the membrane that then reduces enzymatic degradation of the lipid. U73343 was created from U73122 by introduction of a succinimide group in place of the maleimide group, which results in a marked reduction in electrophilicity^35^. Therefore a lower level of accumulation in the anionic inner membrane layer and/or reduced binding to PI(4,5)P_2_ can explain the inability of U73343 to inhibit PLC, and also the lack of effect on the DMS at the same concentration as U73122 (Fig. 6). Further studies are required to support this proposal, such as expression of molecular tools that allow experimental depletion of PI(4,5)P_2_ or detect interference of PH domain binding by CADs. Schulze and colleagues^17^ have previously used the fluorescently tagged PH domain of PLCδ1 to demonstrate that PI(4,5)P_2_ is localised to the DMS in late stage megakaryocytes. However, the DMS collapse induced by CADs will cause redistribution of this PLCδ1-PH construct regardless of its ability to bind PI(4,5)P_2_. Future studies using megakaryocytes derived *in vitro* will also need to ensure that the properties of the DMS within cultured cells match those of the native tissue. In our initial studies of standard megakaryocyte culture systems, membrane capacitance measurements indicated considerably lower levels of DMS in cultured megakaryocytes compared to similar size or even smaller cells obtained directly from the marrow (Gwen Tolhurst and Martyn Mahaut-Smith, unpublished observations).

In contrast to the direct suggestion of a role for PI(4,5)P_2_ in DMS formation, the importance of this lipid in T tubule formation is unclear. However, in myotonic dystrophy, altered skeletal muscle T tubule formation results from expression of a mispliced variant of Bin1 that loses its ability to bind to phosphoinositides^49^. Furthermore, in cardiac myocytes PI(4,5)P_2_ shows a periodicity that coincides with T tubule location^50^ and one brief report suggests that PI(4,5)P_2_ depletion leads to loss of T tubules^51^. It is known that Bin1 localizes to the T tubules in cardiac myocytes and therefore it is likely that PI(4,5)P_2_ contributes to the function of this scaffolding protein in the heart, including tubule morphology and optimal positioning of voltage-gated Ca^2+^ channels near ryanodine receptors^45^,^52^,^53^.

Phenothiazines such as trifluoperazine and chlorpromazine are used as anti-psychotics on the basis of their action at D2 receptors^54^. A variety of side effects have been described for these drugs, including thrombocytopenia^30^,^31^,^32^ and altered cardiac function^55^,^56^,^57^,^58^. Plasma concentrations of phenothiazines following standard clinical use vary with the specific drug but normally reach levels far less than those used in our study (eg. up to ≈0.5 μM for chlorpromazine)^59^, however, they are administered at considerably higher doses in some patients^60^. In addition, phenothiazines have been shown to accumulate in tissues thus the actual concentration that various cell types will experience is unclear^61^,^62^. It is therefore possible that membrane detubulation may explain some of the reported clinical side-effects of CADs^38^, although further studies are required to address this point. Binding to PI(4,5)P_2_ by such drugs may also interfere with other cellular roles dependent upon this phosphoinositide such as endocytosis, exocytosis and regulation of ion channels and transporters^40^,^63^,^64^,^65^. All of the reagents shown in this study to induce detubulation are also established inhibitors of platelet function^66^,^67^, via various proposed targets. These include inhibition of PLC for U73122^35^ or calmodulin for W-7^68^. Our study extends the inhibitory targets for these drugs to loss of the OCS, which has a key role in platelet granule secretion and shape change^21^,^22^,^23^.

In conclusion, this study provides evidence for a key role of negatively charged lipids in the stability of specialised membrane invagination systems such as muscle T tubules and megakaryocyte demarcation membranes. The ability of cationic amphiphiles to detach T tubules and the DMS from the cell surface provides a useful tool for the study of these surface invagination systems, however it should also be considered in the design of CADs as therapeutic treatments.

## Methods

### Reagents

U73343 and U73122 were from Tocris Bioscience (Bristol, UK). Staurosporine was from Enzo Life Sciences (Exeter, UK). KT 5720 was from Santa Cruz Biotechnology (Heidelberg, Germany). Jasplakinolide and LIM kinase inhibitor 1 LIMKi3 were from Calbiochem (Nottinghamshire, UK). FM 1-43 was from Invitrogen (Paisley, UK). Glutaraldehyde, 25% EM Grade was from Agar Scientific (Essex, UK). All other reagents, including HPTS were from Sigma-Aldrich (Dorset, UK).

### Cell preparation

M**eg**akaryocytes and ventricular myocytes were isolated from adult male Wistar rats (300-400 g) following euthanasia in accordance with the UK Animals Scientific Procedures Act. Cells were used within 12 hours of dispersal from the native tissue. Femoral and tibial marrow cells were extracted as described in detail elsewhere^28^ and megakaryocytes distinguished from other marrow cells by their large size, approximately 15–45 μm in diameter, in addition to a multilobular nucleus. Normal physiological saline (NPS), used in all experiments on living megakaryocytes, consisted of 145 mM NaCl, 5 mM KCl, 1 mM MgCl_2_, 1 mM CaCl_2_, 10 mM HEPES, 10 mM glucose, pH 7.35 with NaOH. Ventricular myocytes were prepared using retrograde perfusion via a Langendorff cannula as described in detail elsewhere^69^. Briefly, the heart was rapidly excised, placed into cold Ca^2+^-free Tyrode’s solution (0CaTS; 5 mM KCl, 135 mM NaCl, 0.33 mM NaH_2_PO_4_, 5 mM Na Pyruvate, 10 mM HEPES, 15 mM mannitol, 5 mM glucose, 1 mM MgCl_2_) and rapidly cannulated via the aorta. After perfusion for 6 minutes with 0CaTS, the perfusate was switched to 0CaTS with an enzyme mix (2.5 mg/ml BSA, 0.5 mg/ml collagenase, 0.11 mg/ml protease and 0.09 mg/ml hyaluronidase) and then exchanged for 2 mM Ca^2+^ Tyrode’s solution (2CaTS) on identification of rod-shaped cardiomyocytes. The heart was then cut down, and cardiomyocytes isolated from the tissue in a shaking water bath. Cells were resuspended in fresh 2CaTS, filtered to remove undigested tissue and resuspended in fresh 2CaTS. For confocal recordings, myocytes were immersed into NPS with 1 mM EGTA that had an estimated free Ca^2+^ of 50 nM (MaxChelator, C.Patton, Stanford, USA) to avoid spontaneous contractions, prior to addition of FM 1–43 (8 μM). Washed human platelets were prepared using a previously established method that minimizes spontaneous activation^70^. Human blood was taken from informed, consenting donors as approved by the University of Leicester Human Biology Ethics Committee (non NHS). Blood was mixed with acid citrate anticoagulant (85 mM trisodium citrate, 78 mM citric acid, 111 mM glucose) at a ratio of 6:1 v/v. This mixture was centrifuged at 700 *g* for 5 minutes to obtain platelet-rich plasma (PRP) which was treated with apyrase (0.32 U/ml) and aspirin (100 μM) to reduce spontaneous activation by released nucleotides and thromboxaneA_2_, respectively. Washed platelet suspensions were prepared by centrifugation of PRP at 350 *g* for 20 minutes and resuspension in nominally Ca^2+^-free NPS containing 0.32 U/ml apyrase but without aspirin. Human erythroleukaemia (HEL) cells were sourced and cultured as described elsewhere^25^.

### Fluorescence confocal microscopy

Confocal fluorescence imaging was conducted on an Olympus IX81 inverted microscope equipped with a FluoView1000 laser scanning module (Olympus, UK). All experiments used a 60x oil immersion lens (UPLSAPO 60x, NA 1.35) and a confocal slice thickness of 1.25 μm. Confocal images were visualized and analysed using ImageJ software (Rasband, W.S., ImageJ, U. S. National Institutes of Health, Bethesda, Maryland, USA, http://imagej.nih.gov/ij/, 1997–2012). In order to avoid uptake of fluorescent indicators by endocytosis, all experiments were conducted at room temperature and measurements made within 5 min of exposure to the fluorophore. For wash experiments, the reagents were removed by multiple exchange of the extracellular medium and indicators added after 2 or 120 minutes. HPTS was excited at 488 nm and emission collected at 500–600 nm. FM 1–43 (8 μM) was excited at 488 nm and emission collected at 550–650 nm.

### Quantification of the DMS and T tubules from fluorescence images

We used standard confocal fluorescence imaging to measure entry of an extracellular impermeant fluorescence indicator (HPTS, 400 μM) into tubules of the DMS as a means of quanitifying this plasma membrane invagination system. This approach represents an extension of previous work with 2-photon fluorescence microscopy^25^.The average background-corrected fluorescence within a whole-cell region of interest (ROI) was expressed as a percentage of the average background-corrected fluorescence within an equivalent size ROI in the extracellular fluid (ECF), *i.e.* F_CellROI_/F_ECFROI_ × 100. For each sample, multiple ECF ROI measurements were averaged to avoid heterogeneity. The background value was obtained from the same regions of interest at the same microscope gain settings without added dye. T tubules within cardiac myocytes were quantified from the background-corrected FM 1–43 fluorescence within a ROI drawn within the cell boundary that excluded the peripheral plasma membrane (Sub-PM FM 1–43 fluorescence).

### Transmission electron microscopy

After treatment with reagent or vehicle control, suspensions of marrow cells or human platelets were fixed for 60 minutes in 1.25% glutaraldehyde in 0.1 M sodium cacodylate (0.1 M sodium cacodylate, 2% w/v sucrose, 1 mM CaCl_2_, 1 mM MgCl_2_, pH 7.35 with HCl), washed 3 times and postfixed for 90 minutes in 1% aqueous osmium tetroxide with 1.5% potassium ferricyanide. After further washing, the samples were stained for 60 minutes in 1% aqueous uranyl acetate, washed, pelleted, and embedded in 3% agar gel. Once set, the agar pellets were cut into 1 mm cubes, serially dehydrated by ethanol, and then transferred through a series of pure ethanol mixed with an increasing concentration of Spurr’s modified resin (Agar Scientific; Essex, UK). After several exchanges in pure resin, the samples were polymerised for 16 hours at 60 °C. Sections of approximately 90 nm thickness were cut using a Reichert Ultracut E ultramicrotome (Reichert Jung, Austria), collected onto copper mesh grids and counterstained for 2 min in Reynold’s Lead citrate. Sections were viewed on a JEM-1400 TEM (JEOL UK) with an accelerating voltage of 80 kV and images captured using a Megaview III digital camera with iTEM software (Olympus Softimaging Solutions; Münster, Germany). The OCS was quantified as the percentage cytoplasmic area occupied by all vacuolar regions within an individual platelet.

### Electrophysiological assessment of the DMS using whole cell membrane capacitance

Conventional whole cell recordings under voltage clamp were performed using an Axopatch 200B patch clamp amplifier (Molecular Devices, CA, USA) under the control of Clampex v6.0 electrophysiological software (Molecular Devices). Currents were low-pass filtered (5 kHz) and acquired at a rate of 50 kHz. Capacitative current transients were recorded in response to 60 ms duration −10 mV voltage steps from a holding potential of −80 mV and membrane capacitance calculated from the integral of the current as described in detail elsewhere^28^. The average cell diameter was assessed from two perpendicular planes and surface area (SA) was calculated assuming a spherical geometry to allow expression of capacitance per area of peripheral surface membrane.

### Statistical analysis

Data were presented as the mean ± SEM and significance was determined using one way ANOVA with Bonferroni post-testing, with the exception of changes in platelet OCS area which was assessed using Student’s unpaired T test with Welch’s correction (GraphPad Prism 6). The level of significance is indicated as not significant (*P *>* 0.05*; ns), or significant at *P *<* 0.05* (*), *P *<* 0.01* (**), *P *<* 0.001* (***) and *P* < *0.0001* (****).

## Additional Information

**How to cite this article**: Osman, S. *et al.* Detachment of surface membrane invagination systems by cationic amphiphilic drugs. *Sci. Rep.* **5**, 18536; doi: 10.1038/srep18536 (2015).

## Supplementary Material

Supplementary Information

Supplementary Movie S1

Supplementary Movie S2

Supplementary Movie S3

Supplementary Movie S4

Supplementary Movie S5

Supplementary Movie S6
