# Supplementary material for: Detachment of surface membrane invagination systems by cationic amphiphilic drugs
Source: Sci Rep. 2016 Jan 4;6:18536. doi: 10.1038/srep18536 (PMC4698757; doi:10.1038/srep18536)
Supplement: Supplementary Information [file srep18536-s1.pdf]

Detachment of surface membrane invagination systems by cationic amphiphilic drugs

Sangar Osman, Kirk A. Taylor, Natalie Allcock, Richard D. Rainbow and Martyn P. Mahaut-Smith

Supplementary information

Figure S1. Entry of extracellular dye into the DMS correlates with megakaryocyte size. Graph shows the relationship between megakaryocyte diameter and the cellular fluorescence ($F_{CellROI}$) arising from an impermeant extracellular indicator (HPTS), expressed as a percentage of the signal within the extracellular fluid (F_{ECFROI}). The diameter was the average of two perpendicular measurements across the cell. Cell that lack DMS show a background percentage fluorescence level within the cellular ROI of 4.67 $\pm$ 0.17, which represents out of focus fluorescence. Data are from 136 megakaryocytes. (r^2=0.46, *P<0.0001*).

Figure S2. Block of extracellular dye access to the DMS by W-7 is reversible and not prevented by a metalloproteinase inhibitor. ai) Transmitted and fluorescence images from megakaryocytes that had been exposed to 150 µM W-7 for 15 min, then washed and immersed in an impermeant extracellular indicator (HPTS) after a further 2 min (left panel) or 120 min (right panel). **aii**) Marrow cells were treated for 15 min with 150 µM W-7 or DMSO, then washed and resuspended in NPS. Cells were immersed in HPTS and the average whole cell fluorescence assessed within 5 min at the end of the W-7 treatment, after 2 min wash, and after 120 min wash for W-7 and DMSO-treated megakaryocytes. **b)** Whole cell fluorescence measured after treatment with the metalloproteinase inhibitor GM6001 (100 µM GM6001) alone or prior to W-7 (100 or 150 µM for 15 min). Scale bars: 10 µm.

Figure S3. Aspirin, an anionic amphiphilic drug, does not interfere with entry of an extracellular indicator into the DMS. a) Transmitted and fluorescence images from megakaryocytes immersed in an impermeant extracellular indicator (HPTS) after exposure to vehicle control (left panel) or aspirin

(right panel). **b)** Quantification of HPTS entry into the megakaryocyte in vehicle-treated (control) versus aspirin-treated cells. Scale bars: 10 µm.

Figure S4. Loss of cardiac myocyte T tubules induced by CADs is not readily reversed. Cardiac myocytes were exposed to vehicle (control), 40 µM trifluoperazine (TFP) or 300 µM imipramine (IMP) for 15 min, washed and resuspended in NPS for 120 min, then exposed FM 1-43. **a)** Sample images of FM1-43 staining. **b)** Average FM 1-43 fluorescence within a region of interest drawn around the periphery of the cell but excluding the peripheral plasma membrane (Sub-PM FM 1-43 fluorescence). Scale bar: 10 µm.

Table S1. Effect of targeting kinases, cytoskeletal proteins and signalling events on DMS tubule staining by an extracellular indicator. Cells were exposed to each reagent at the concentration specified or its vehicle control, for the duration indicated. HPTS was then added to the extracellular medium and the percentage fluorescence within a whole cell region of interest measured relative to the extracellular fluorescence.

Table S2. Effect of targeting kinases, cytoskeletal proteins or signalling events on the ability of W-7 to block extracellular dye access to the DMS. Cells were exposed to each reagent at the concentration specified, or its vehicle control, for 60 min followed by 150 µM W-7 for 15 min. HPTS was then added to the extracellular medium and the percentage fluorescence within a whole cell region of interest measured relative to the extracellular fluorescence

Table S3. Effect of additional cationic or anionic amphiphilic drugs on HPTS entry to the megakaryocyte DMS. Megakaryocytes were exposed to each reagent at the concentration specified or its vehicle control, for 15 min. HPTS was then added to the extracellular medium and the

percentage fluorescence within a whole cell region of interest measured relative to the extracellular fluorescence

Movie S1. W-7-induced megakaryocyte membrane movement. Confocal fluorescence imaging time series for a megakaryocyte stained with the membrane indicator FM 1-43 during exposure to 150 µM W-7. Images were captured every 30 sec for a total of 15 minutes. 8 µM FM 1-43 was added to the chamber ≈5 min prior to the start of the movie. The dye stains the DMS and peripheral plasma membrane, which start to separate at ≈7 min. W-7 was applied to the cell immediately before the start of the movie. Scale bar: 10 µm.

Movie S2. Cardiac myocyte FM 1-43 control. Confocal fluorescence imaging z-series through a cardiac myocyte stained with FM 1-43 under control conditions. Scale bar: 10 µm.

Movie S3. Cardiac myocyte FM 1-43 after W7 treatment. Confocal fluorescence imaging z-series through a cardiac myocyte stained with FM 1-43 after exposure to 100 µM W-7 for 15 min. Scale bar: 10 µm.

Movie S4. Cardiac myocyte FM 1-43 after TFP treatment. Confocal fluorescence imaging z-series through a cardiac myocyte stained with FM 1-43 after exposure to 40 µM trifluoperazine for 15 min. Scale bar: 10 µm.

Movie S5. Cardiac myocyte FM 1-43 after imipramine treatment. Confocal fluorescence imaging z-series through a cardiac myocyte stained with FM 1-43 after exposure to 300 µM imipramine for 15 min. Scale bar: 10 µm.

Movie S6. Cardiac myocyte FM 1-43 after verapamil treatment. Confocal fluorescence imaging z-series through a cardiac myocyte stained with FM 1-43 after exposure to 1mM verapamil for 15 min. Scale bar: 10 μm.

Figure S1

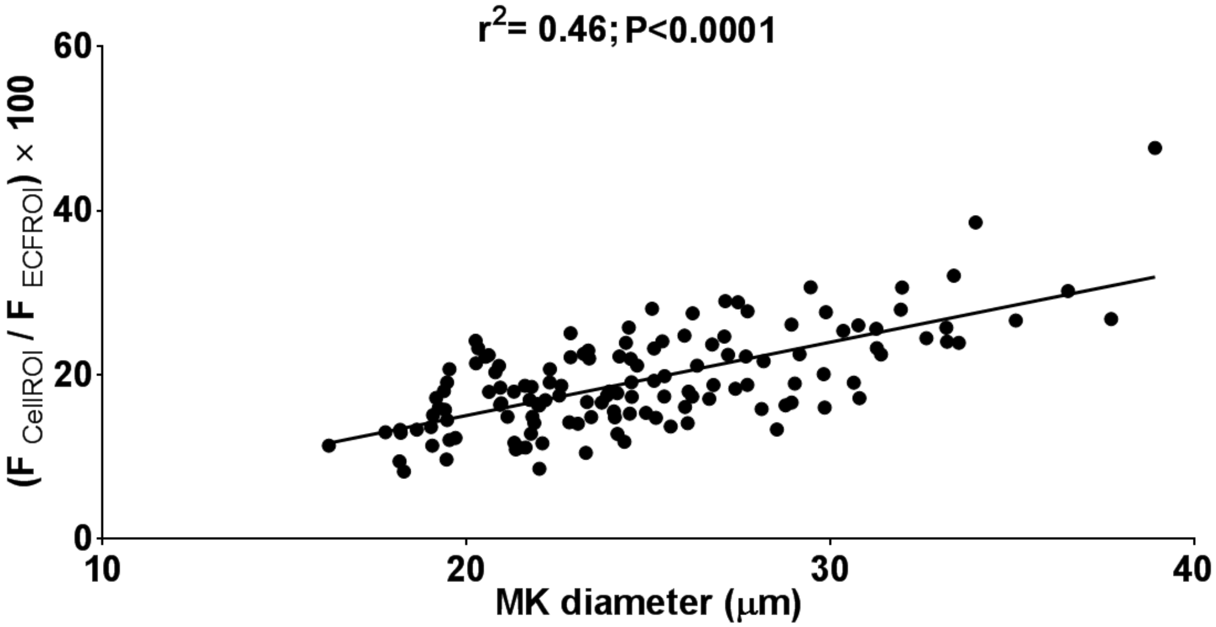

r2= 0.46; P<0.0001
(F CellROI / F ECFROI) × 100
0
20
40
60
10
20
30
40
MK diameter (μm)

Figure S2

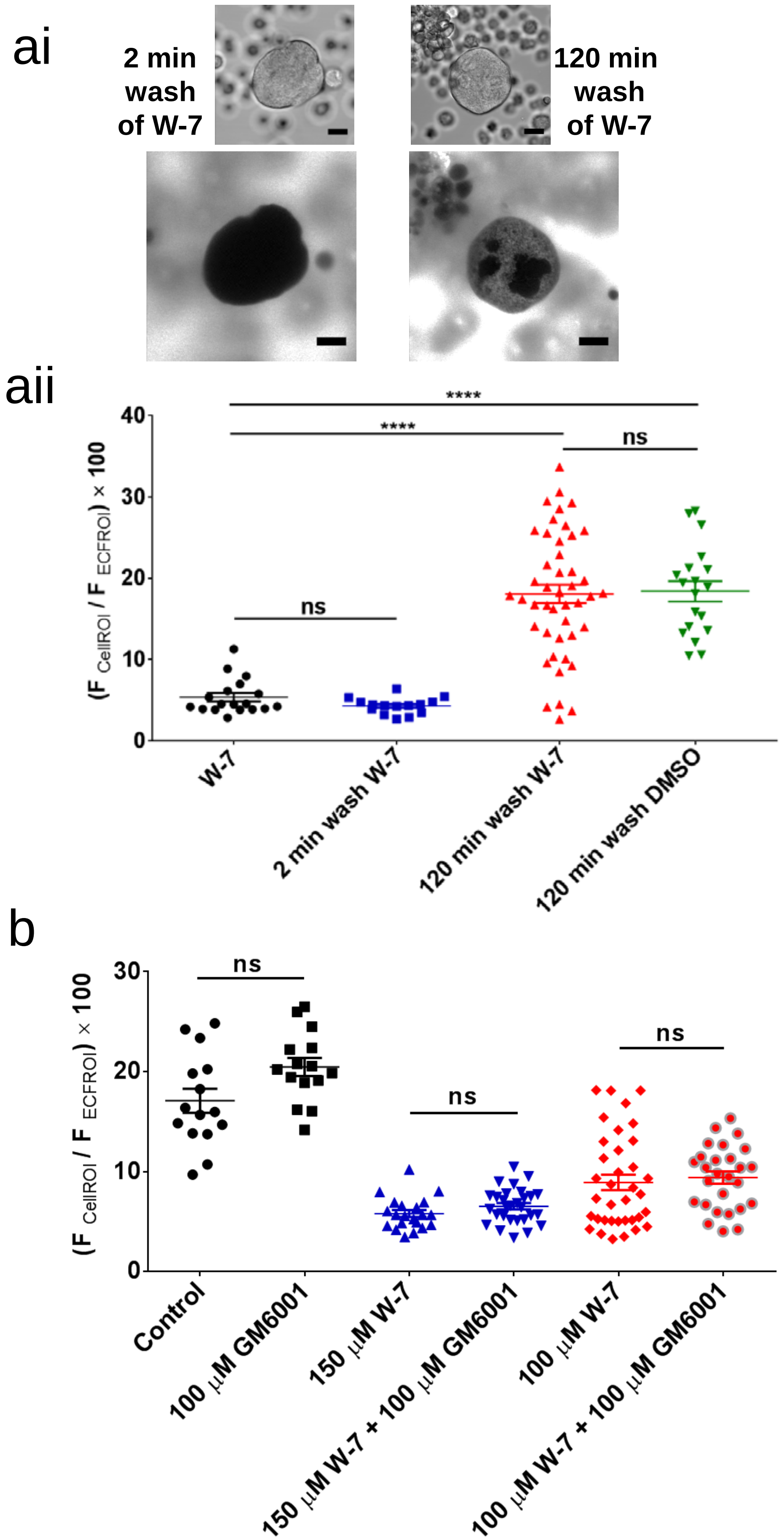
ai
2 min wash of W-7
120 min wash of W-7
aii
(F CellROI / F ECFROI) × 100
****
****
ns
ns
W-7
2 min wash W-7
120 min wash W-7
120 min wash DMSO
b
(F CellROI / F ECFROI) × 100
ns
ns
ns
Control
100 µM GM6001
150 µM W-7
150 µM W-7 + 100 µM GM6001
100 µM W-7
100 µM W-7 + 100 µM GM6001

Figure S3

a

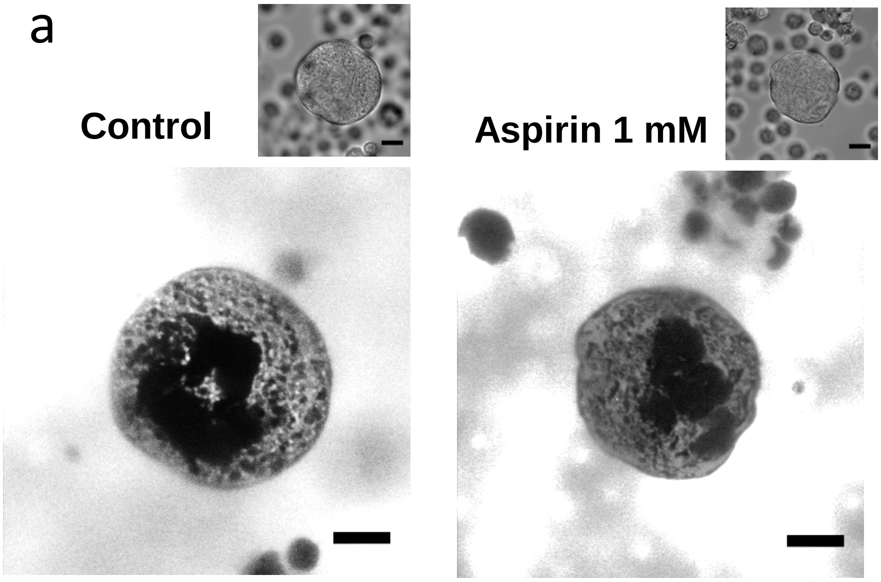
Control
Aspirin 1 mM

b

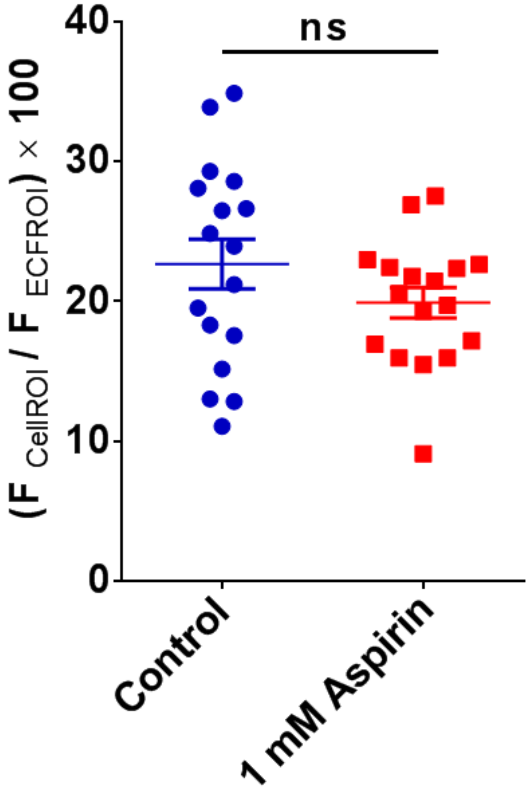
ns
40
30
20
10
0
(F CellROI / F ECFROI) × 100
Control
1 mM Aspirin

Figure S4

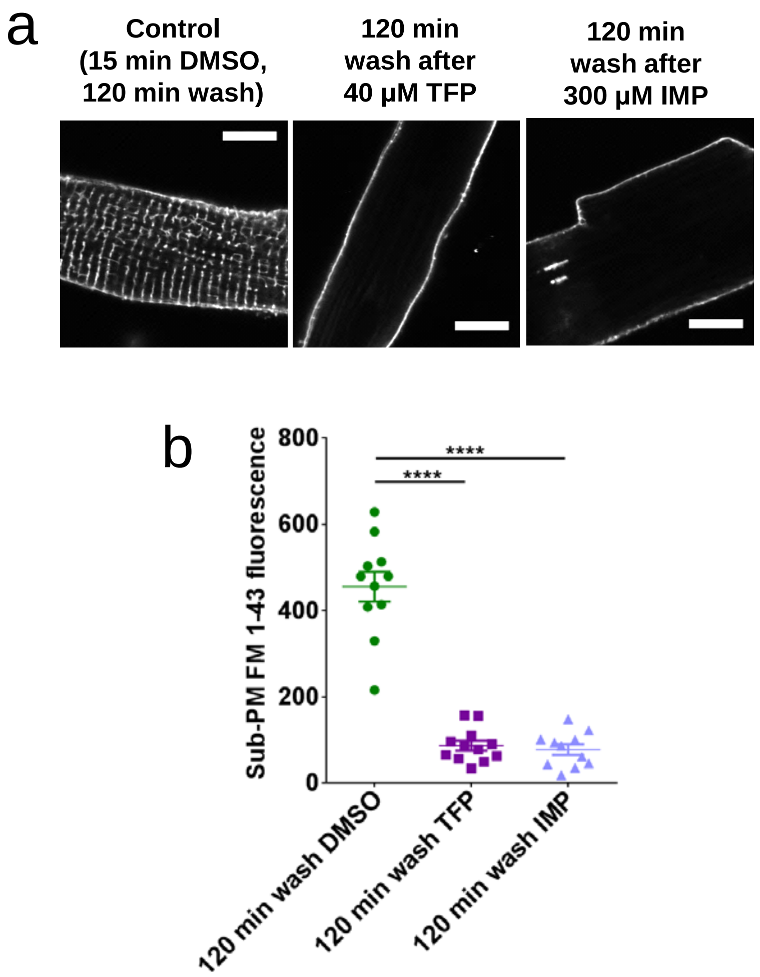
a
Control
(15 min DMSO,
120 min wash)
120 min
wash after
40 μM TFP
120 min
wash after
300 μM IMP
b
Sub-PM FM 1-43 fluorescence
800
600
400
200
0
****
****
120 min wash DMSO
120 min wash TFP
120 min wash IMP

Detachment of surface membrane invagination systems by cationic amphiphilic drugs

Sangar Osman, Kirk A. Taylor, Natalie Allcock, Richard D. Rainbow and Martyn P. Mahaut-Smith

Table S1. Effect of targeting kinases, cytoskeletal proteins and signalling events on DMS tubule staining by an extracellular indicator

Reagent	Known effect	$(F_{CellROI} / F_{ECFROI}) \times 100$
**Protein kinase inhibitors**		
ML-7	Selective inhibitor of myosin light chain kinase	40 µM, 60 min: 19.90 ± 1.37 % (n=20); control: 18.03 ± 1.71 % (n=23); (*P>0.05*)
Akt 1/2 kinase inhibitor	Protein kinase B inhibitor	20 µM, 60 min: 18.10 ±1.65 % (n=20); control: 16.67 ± 1.20 % (n=23); (*P>0.05*)
GF 109203X	Protein kinase C inhibitor	20 µM, 60 min: 20.95 ± 1.05 % (n=11); Control: 20.69 ± 0.85 % (n=23); (*P>0.05*)
PD 98059	Inhibitor of MAPKK (MKK / MEK)	50 µM, 60 min: 21.22 ± 1.04 % (n=29); Control: 19.75 ± 0.88 % (n=33); (*P>0.05*)
KN-93	CaM kinase II	20 µM, 60 min: 19.06 ± 0.95 % (n=16); Control: 20.06 ± 0.92 % (n=22); (*P>0.05*)
Anagrelide hydrochloride	Potent type III phosphodiesterase (PDE3) inhibitor	10 µM, 60 min: 18.16 ± 1.05 % (n=18); Control: 19.26±0.62 % (n=20); (*P>0.05*)
Staurosporine	Broad spectrum protein kinase inhibitor (mainly PKC)	1 µM, 60 min: 19.36 ± 1.65 % (n=22); Control: 19.24 ± 0.90 % (n=28); (*P>0.05*)
Genistein	Inhibitor of tyrosine protein kinase	150 µM, 60 min: 17.80 ± 2.21 % (n=17); Control: 16.93 ± 1.75 % (n=18); (*P>0.05*)
LY 294002 hydrochloride	PI_3K inhibitor	50 µM, 60 min: 20.27 ± 1.17 % (n=18); Control: 18.51 ± 1.49 % (n=15); (*P>0.05*)
TBCA	Casein kinase CK2 inhibitor	25 µM, 60 min: 19.27 ± 0.77 % (n=16); Control: 20.62 ± 0.89 % (n=15); (*P>0.05*)
LIM kinase inhibitor I	Potent LIM kinase inhibitor	20 µM, 60 min: 19.29 ± 1.18 % (n=15); Control: 19.82 ± 1.12 % (n=20); (*P>0.05*)
KT 5720	Protein kinase A inhibitor	10 µM, 60 min: 17.33 ±1.22 % (n=14); Control: 18.62 ± 0.32 % (n=15); (*P>0.05*)
**Cytoskeleton & Motor protein inhibitors**		
(-)-Blebbistatin	Selective inhibitor of non-muscle myosin II	100 µM, 60 min: 18.36 ± 1.14 % (n=25); Control: 19.96 ± 1.29 % (n=19); (*P>0.05*)
Paclitaxel	Stabilizes microtubules	50 µM, 60 min: 20.16 ± 0.98 % (n=21); Control: 18.65 ± 0.69 % (n=16); (*P>0.05*)
Jasplakinolide	Stabilizes pre-formed actin filament	200 nM, 60 min: 19.98 ± 1.34 % (n=19); Control: 20.58 ± 1.09 % (n=21); (*P>0.05*)

Cytochalasin D	Cell permeable actin depolymerisation agent	5 µM, 60 min: 19.35 ± 1.23 % (n=15); Control: 16.97 ± 0.89 % (n=21); (*P>0.05*)
Nocodazole	Cell permeable microtubule disrupting agent	10 µM, 60 min: 16.77 ± 0.93 % (n=15); Control: 16.97 ± 1.05 % (n=21); (*P>0.05*)
**<u>Agents that cause glycoprotein ectodomain cleavage</u>**		
CCCP	Uncoupler of oxidative phosphorylation: induces ectodomain cleavage by mimicking platelet ageing	100 µM, 60 min: 20.84 ± 1.21 % (n=17); Control: 20.92 ± 0.85 % (n=23); (*P*>0.05)
N-ethylmaleimide	Thiol-modifying reagent: directly activates metalloproteinases	2 mM, 60 min: 21.58 ± 1.01 % (n=15); Control: 19.59 ± 1.24 % (n=15); (P>0.05)
**<u>Modulation of signalling events</u>**		
ADPβS	P2Y receptors agonist	100 µM, 5 min: 19.66 ± 0.22 % (n=5); Control: 18.16 ± 0.95 % (n=5); (*P>0.05*)
5,5′-Dimethyl-BAPTA-AM	Intracellular calcium chelator	50 µM, 60min: 18.74 ± 0.97 % (n= 21); Control: 20.86 ±1.23 % (n= 20); (*P>0.05*)
TPEN	High-affinity chelator of heavy metals (Zn^{2+}>Fe^{2+}>Mn^{2+}>>Ca^{2+}=Mg^{2+})	100 µM, 45 min: 19.14 ± 1.39 % (n= 15); Control: 16.86 ± 1.33 % (n= 15); (*P>0.05*)
Ionomycin	Mobile ion carrier for Ca^{2+}	1 µM, 5 min: 18.28 ± 1.76 % (n=15); Control: 21.58 ± 1.81 % (n= 15); (*P>0.05*)

Table S2. Effect of targeting kinases, cytoskeletal proteins or signalling events on the ability of W-7 to block extracellular dye access to the DMS

Reagent	Known effect	($F_{CellROI}$ / F_{ECFROI}) × 100
**Modulation of signalling events**		
5,5′-Dimethyl-BAPTA-AM	Intracellular calcium chelator	50 µM: 4.32 ± 0.91 % (n= 20);<br>Control: 4.94 ± 0.86 % (n= 20); (*P>0.05*)
TPEN	High-affinity chelator of heavy metals (Zn^{2+}>Fe^{2+}>Mn^{2+}>>Ca^{2+}=Mg^{2+})	100 µM: 6.40 ± 0.67 % (n= 29);<br>Control: 5.63 ± 0.47 % (n= 15); (*P>0.05*)
Ionomycin	Mobile ion carrier for Ca^{2+}	1 µM: 5.01 ± 1.07 % (n= 15);<br>Control: 4.33 ± 1.66 % (n=15); (*P>0.05*)
**Protein kinase inhibitors**		
ML-7	Selective inhibitor of myosin light chain kinase	40 µM: 3.48 ± 0.14 % (n=15);<br>Control: 3.47 ± 0.22 % (n= 15); (*P>0.05*)
Akt 1/2 kinase inhibitor	Protein kinase B inhibitor	20 µM: 2.52 ± 0.37 % (n= 15);<br>Control: 3.81 ± 0.11 % (n= 19); (*P>0.05*)
GF 109203X	Protein kinase C inhibitor	20 µM: 5.68 ± 0.34 % (n= 16);<br>Control: 3.78 ± 0.14 % (n= 20); (*P>0.05*)
PD 98059	Inhibitor of MAPKK (MKK / MEK)	50 µM: 3.39 ± 0.34 % (n= 14);<br>Control: 3.42 ± 0.16 % (n= 16); (*P>0.05*)
KN-93	CaM kinase II	20 µM: 4.27 ± 0.20 % (n= 16);<br>Control: 3.66 ± 1.16 % (n= 16); (*P>0.05*)
Anagrelide hydrochloride	Potent type III phosphodiesterase (PDE3) inhibitor	10 µM: 5.17 ± 0.83 % (n= 15);<br>Control: 4.97 ± 1.06 % (n= 15); (*P>0.05*)
Staurosporine	Broad spectrum protein kinase inhibitor (mainly PKC)	1 µM: 3.126 ± 0.20 % (n= 16);<br>Control: 4.57 ± 0.28 % (n= 16); (*P>0.05*)
LY 294002 hydrochloride	PI_3K inhibitor	50 µM: 4.60 ± 0.47 % (n= 15);<br>Control: 3.59 ± 0.56 % (n= 15); (*P>0.05*)
LIM kinase inhibitor I	Potent LIM kinase inhibitor	20 µM: 3.18 ± 0.56 % (n= 15);<br>Control: 3.93 ± 0.26 % (n= 16); (*P>0.05*)
KT 5720	Protein kinase A inhibitor	10 µM: 5.08±1.34 % (n= 15);<br>Control: 5.33±1.06 % (n= 16); (*P>0.05*)
**Cytoskeleton & Motor protein inhibitors**		
(-)-Blebbistatin	Selective inhibitor of non-muscle myosin II	100 µM: 4.57 ± 0.29 % (n= 14);<br>Control: 3.47 ± 0.22 % (n= 14); (*P>0.05*)
Paclitaxel	Stabilizes microtubules	50 µM: 3.77 ± 1.09 % (n= 15);<br>Control: 5.11±1.14 % (n= 15); (*P>0.05*)
Jasplakinolide	Stabilizes pre-formed actin filament	200 nM: 4.02 ± 0.26 % (n= 29);<br>Control: 3.82 ± 0.22 % (n= 24); (*P>0.05*)

Table S3. Effect of additional cationic or anionic amphiphilic drugs on HPTS entry to the megakaryocyte DMS

**Amphiphilic drug (anionic or cationic)**	**Established action**	**$(F_{CellROI} / F_{ECFROI}) \times 100$**
Imipramine hydrochloride (IMP)<br>(cationic)	Tricyclic antidepressant agent	100 µM: 25.44 ± 1.30 % (n= 13);<br>Control: 20.97 ± 0.91 % (n= 30); (*P>0.05*)<br>200 µM: 15.88 ± 1.42 % (n= 17);<br>Control: 22.28 ± 1.30 % (n= 15); (*P>0.05*)<br>300 µM: 8.95 ± 0.95 % (n= 28);<br>Control: 23.49 ± 1.47 % (n= 24); (*P<0.0001*)<br>400 µM: 6.26 ± 0.57 % (n= 36);<br>Control: 24.68 ± 0.89 % (n= 21); (*P<0.0001*)
Verapamil hydrochloride (VPM)<br>(cationic)	Calcium channel blocker	200 µM: 20.87 ± 0.74 % (n= 21);<br>Control: 18.25 ± 1.30 % (n= 20); (*P>0.05*)<br>500 µM: 18.40 ± 1.05 % (n= 24);<br>Control: 17.75 ± 1.46 % (n= 15); (*P>0.05*)<br>750 µM: 5.48 ± 0.66 % (n= 27);<br>Control: 20.83 ± 1.31 % (n= 18); (*P<0.0001*)<br>900 µM: 4.67 ± 0.78 % (n=25);<br>Control: 19.97 ± 0.85 % (n= 22); (*P<0.0001*)<br>1 mM: 4.43 ± 0.27 % (n= 29);<br>Control: 20.84 ± 0.87 % (n= 30); (*P<0.0001*)
Propranolol hydrochloride (PRO)<br>(cationic)	β-adrenergic blocker	200 µM: 19.46 ± 0.78 % (n= 19);<br>Control: 20.81 ± 1.08 % (n= 24); (*P>0.05*)<br>400 µM: 15.24 ± 1.02 % (n= 23);<br>Control: 19.76 ± 0.90 % (n= 24); (*P<0.01*)<br>600 µM: 4.70 ± 0.35 % (n= 38);<br>Control: 21.23 ± 0.54 % (n= 46); (*P<0.0001*)<br>800 µM: 3.58 ± 0.67 % (n= 30);<br>Control: 20.46 ± 0.85 % (n= 30); (*P<0.0001*)
Bupivacaine hydrochloride (BUP)<br>(cationic)	Local anesthetic	1 mM: 18.20 ± 1.07 % (n= 21);<br>Control: 19.16 ± 1.12 % (n= 20); (*P>0.05*).<br>5 mM: 12.32 ± 1.32 % (n= 22);<br>Control: 20.33 ± 0.84 % (n= 16); (*P<0.01*)<br>8 mM: 8.11 ± 0.87 % (n= 25);<br>Control: 18.13 ± 1.14 % (n=19); (*P<0.0001*)<br>10 mM: 6.34 ± 0.47 %, (n= 38);<br>Control: 21.16 ± 0.73 % (n= 22); (*P<0.0001*)
Sodium dodocyl sulfate (SDS)<br>(anionic)	Anionic detergent	150 µM: 16.65 ± 1.14 % (n=12);<br>Control: 19.71 ± 0.97 % (n=15); (*P>0.05*)
Sodium thiopental (THI)	Short-acting barbiturate	300 µM: 22.55 ± 1.25 % (n=14);

(anionic)	general anesthetic	Control: 23.62 ± 1.73 % (n=12); (*P>0.05*)<br>500 μM: 18.26 ± 1.88 % (n=16);<br>Control: 17.31 ± 1.17 % (n=15); (*P>0.05*)
